# Bovine Leukemia Virus: Global Prevalence and Localized Resurgence in China Revealed by an Integrated Meta‐Analysis and Case Series

**DOI:** 10.1155/tbed/2598815

**Published:** 2026-04-10

**Authors:** Ruicheng Yu, Sen Zhang, Zhijie Xiang, Liyun Wan, Marawan A. Marawan, Jianguo Chen, Aizhen Guo, Yingyu Chen

**Affiliations:** ^1^ National Key Laboratory of Agricultural Microbiology, College of Veterinary Medicine, Huazhong Agricultural University, Wuhan, 430070, China, hzau.edu.cn; ^2^ Hubei International Scientific and Technological Cooperation Base of Veterinary Epidemiology, The Cooperative Innovation Center for Sustainable Pig Production, Wuhan, 430070, China; ^3^ Key Laboratory of Development of Veterinary Diagnostic Products, Ministry of Agriculture and Rural Affair, Wuhan, 430070, China, agri.gov.cn; ^4^ Animal Medicine Department (infectious diseases), Faculty of Veterinary Medicine, Benha University, Toukh, 44629, Egypt, bu.edu.eg; ^5^ Hubei Hongshan Laboratory, Hubei Jiangxia Laboratory, Wuhan, 430070, China, hzau.edu.cn

**Keywords:** bovine leukemia virus, case series, china, enzootic bovine leukosis, global prevalence, localized resurgence, meta-analysis

## Abstract

**Background:**

Bovine leukemia virus (BLV), the causative agent of enzootic bovine leukosis (EBL), causes substantial economic losses in the global cattle industry. While some regions have made progress in terms of control, the global epidemiological landscape and situation in China have not been fully elucidated. This study aimed to clarify the current global prevalence of BLV based on existing reported studies and investigate its alarming resurgence in China.

**Methods:**

We conducted an integrated study comprising a systematic review and meta‐analysis of the global literature from 2010 to 2025 and a detailed case series investigation from Hubei Province, China. The meta‐analysis followed PRISMA guidelines to calculate the pooled prevalence and identify risk factors. The case series included pathological examination, histopathology, and molecular detection (real‐time PCR and nested PCR) to confirm BLV infection and genotype in clinical cases.

**Results:**

The meta‐analysis, which included 44 studies, revealed a global pooled BLV prevalence of 23.24%, with a significant decline observed in studies published after 2018 (4.35%) compared with those published before 2018 (20.31%). Subgroup analyses indicated marked differences in detection rates across sample types and detection methods. Besides, significantly greater prevalence was also observed in cattle more than 12 months. In stark contrast to this global trend, the pooled prevalence in China was 16.80%, which masked a severe high‐burden endemicity in Hubei Province, where a 49.41% positivity rate (126/255) was detected across three farms. Pathological and molecular analyses of four clinical cases from these farms confirmed BLV‐induced lymphosarcoma and identified the circulating virus as genotype G6.

**Conclusion:**

This study delineates an apparent decline in reported BLV prevalence globally, contrasted with a severe localized resurgence in Hubei Province, China: an apparent decline in reported prevalence based on the available data in BLV prevalence, alongside a severe and ongoing resurgence in a major Chinese cattle‐farming region. These findings underscore that national and global averages can obscure dangerous local epidemics. The situation in Hubei serves as a crucial warning, demanding immediate, targeted interventions, including enhanced surveillance, stringent biosecurity, and farmer education, to curb the spread of BLV.

## 1. Introduction

Bovine leukemia virus (BLV) is a deltaretrovirus that infects cattle, causing persistent infection in the majority of hosts [1]. In ~30% of infected animals, the infection progresses to persistent lymphocytosis, and in 1%–5% of cases, it culminates in fatal B‐cell lymphosarcoma, a condition known as enzootic bovine leukosis (EBL) [2]. Clinical manifestations of EBL include weight loss, depression, lymphadenopathy, and organ failure due to tumor infiltration [3]. BLV is transmitted horizontally via the transfer of infected lymphocytes through iatrogenic means (e.g., contaminated surgical instruments, needles) and vertically from the dam to the calf in utero or through colostrum and milk [4–6].

This disease poses a major economic threat through reduced milk production, premature culling, mortality, and trade restrictions. Previous studies have suggested a potential association between BLV infection and human breast cancer, indicating that this possible link warrants greater attention from a public health perspective [7]. In China, BLV was first identified in 1974 and had since persisted at low prevalence levels. Recent reports suggest a worrying localized resurgence, with the prevalence in some regions rebounding to nearly 50% [8, 9].

While systematic reviews offer valuable pooled prevalence estimates, they can obscure critical local variations and emerging hotspots. This study employs a dual‐strategy approach: first, a comprehensive meta‐analysis to quantify the global and national (Chinese) prevalence of BLV infection and identify key epidemiological risk factors; second, a detailed case series investigation from Hubei Province, China, designed to ground‐truth the meta‐analytical findings and provide clinical, pathological, and molecular evidence of active disease transmission and localized resurgence. This integrated methodology seeks to elucidate BLV epidemiological dynamics and quantify regional disease burdens. Ultimately, these insights will facilitate the design of targeted interventions in specific regions and localities to mitigate the impact of BLV on bovine health and productivity.

## 2. Materials and Methods

### 2.1. Meta‐Analysis

#### 2.1.1. Search Strategy

This meta‐analysis was conducted in accordance with the PRISMA guidelines [10]. To identify studies reporting on the prevalence of BLV or EBL, we systematically searched four electronic databases: PubMed, Web of Science, China National Knowledge Infrastructure (CNKI), and the Wanfang Database. The search encompassed literature published from January 2010 to March 2025. Our search strategy employed a combination of keywords and synonymous terms related to the disease (e.g., “Enzootic bovine leukosis” [Title/Abstract], or “Bovine leukemia virus” [Title/Abstract], or “EBL” [Title/Abstract], or “BLV” [Title/Abstract]) and its epidemiology (e.g., “Prevalence” [Title/Abstract], or “Seroprevalence” [Title/Abstract], or “Outbreak” [Title/Abstract]), limits: publication date from January 01, 2010 to June 30, 2025. To ensure a comprehensive search and minimize selection bias, all database searches were performed independently by two authors (Sen Zhang and Ruicheng Yu), and the reference lists of the retrieved articles were manually screened for additional relevant publications.

#### 2.1.2. Study Selection and Eligibility Criteria

The literature selection process was conducted in strict accordance with the PRISMA guidelines and is summarized in the flow diagram. Following the initial search, all the retrieved records were collated, and duplicates were removed. The study selection was then performed independently by two reviewers (Sen Zhang and Ruicheng Yu) in a two‐stage process to minimize bias.

First, the reviewers screened the titles and abstracts of all unique records against the predefined eligibility criteria. Studies were excluded at this stage if they were (i) review articles, conference abstracts, or editorials; (ii) focused on unrelated topics or other pathogens; (iii) conducted in species other than cattle; or (iv) not primary prevalence studies.

The full texts of the remaining potentially eligible articles were subsequently thoroughly assessed. A study was included in the final meta‐analysis only if it met all of the following criteria: (i) presented original data from an observational study (only cross‐sectional studies were included, cohort or case‐control studies were not taken into consideration) on BLV/EBL prevalence in cattle; (ii) the study population was clearly defined; (iii) the diagnostic method (ELISA for serological diagnosis and PCR for molecular diagnosis) was specified; and (iv) provided sufficient raw data to calculate the prevalence (i.e., the number of positive samples and the total sample size). Studies were excluded from the full‐text review for the following reasons: (i) inability to extract or calculate prevalence data; (ii) irrelevant data or outcomes; (iii) duplicate dataset (when duplicate papers were retrieved from different databases, the most comprehensive or recent publication was retained after approval by both reviewers); (iv) sample size smaller than 50 individuals (aim to reduce instability of extreme proportions); or (v) full text was unavailable despite exhaustive efforts. It should be noted that during the review process, we noticed two papers with different titles and abstracts but with exactly the same content. After discussion between the two reviewers (Sen Zhang and Ruicheng Yu) and a senior author (Yingyu Chen), it was decided to keep the most recently published one.

Any disagreement between the two reviewers regarding the inclusion or exclusion of a study at either stage was resolved through discussion until a consensus was reached. If a consensus could not be reached, a third senior author (Yingyu Chen) was consulted to make the final decision.

#### 2.1.3. Data Extraction and Quality Assessment

A predesigned data extraction form was developed in Microsoft Excel to ensure consistent and accurate data collection from the included studies. The data extraction process was carried out independently by two reviewers (Sen Zhang and Ruicheng Yu). The following information was systematically extracted from each eligible study: (i) basic study information: author and publication year; (ii) geographical details: country and continent of the study; (iii) study methodology: sample type (e.g., serum, whole blood), detection method (e.g., ELISA, PCR), and total sample size; (iv) outcome data: the number of positive samples for BLV/EBL to calculate the prevalence; and (v) subgroup data: where available, raw data were also extracted for prespecified subgroups, including cattle breed (dairy vs. beef), age group (>12 months vs. ≤ 12 months), and sex.

Any discrepancies in the extracted data between the two reviewers were identified and resolved through reexamination of the original article and discussion until a consensus was reached. If necessary, a third author (Yingyu Chen) was consulted for arbitration.

The methodological quality of each included study was assessed independently by the same two reviewers via the Newcastle–Ottawa scale (NOS), adapted for cross‐sectional studies, as described in the methodological literature [11] and applied in recent meta‐analyses in the veterinary field [12]. The scale evaluates studies across three domains: selection (representativeness of the sample, sample size, nonrespondents, and ascertainment of the exposure), comparability (controlling for confounding factors), and outcome (assessment of the outcome and statistical test). Studies were awarded stars for each item, with a maximum score of 10. On the basis of the total score, studies were categorized as high quality (8–10 scores), moderate quality (6–7 scores), or low quality (≤5 scores).

#### 2.1.4. Statistical Synthesis and Analysis

All the statistical analyses were performed via Stata software (Version 17.0; Stata Corporation, College Station, TX, USA), and the meta‐analysis (based on raw proportions) was completed with metaprop command. The pooled prevalence of BLV infection with 95% confidence intervals (CIs) was calculated. Given the anticipated clinical and methodological diversity across studies, a random effects model (DerSimonian and laird method) was employed as the primary analytical framework to provide a more conservative and generalizable estimate. Heterogeneity among the included studies was quantified via the *I* ^2^ statistic, with an *I* ^2^ value >50% considered to represent substantial heterogeneity. The results are presented in a forest plot.

To explore potential sources of heterogeneity and investigate the associations of key factors associated with BLV prevalence, we conducted prespecified subgroup analyses based on the following factors: region, publication year, sample type, detection method, breed, sex, and age. The pooled prevalence rates for each subgroup were calculated using univariate analysis based on the original data, without adjustment for other covariates. To investigate the independent temporal and spatial effects of the reported cases with a marked reduction in prevalence, univariate meta‐regression analyses were also performed. To identify independent predictors of the prevalence and control for potential confounding factors, the multivariate meta‐regression analyses were conducted, and variables that demonstrated potentially statistical significance in subgroup analyses were included in the regression model. Besides, the influence of each study on the overall pooled estimate was assessed via leave‐one‐out sensitivity analysis.

For all analyses, a *p* value <0.05 was considered statistically significant, except for Cochran’s *Q* test for heterogeneity, where a significance level of *p*  < 0.10 was applied.

### 2.2. Case Series Investigation

#### 2.2.1. Patient History and Sample Collection

Case investigations were conducted between March and December 2024 on three cattle farms in Hubei Province, China. The study involved one beef cattle farm (Farm A, 700 Simmental cattle) and two dairy farms (Farm B, 500 Holstein cattle and Farm C, 2500 Holstein cattle). This animal experiment was approved by the Animal Experiment Ethics Committee of Huazhong Agricultural University (Approval Number: HZAUCA‐2024‐0036). All procedures were conducted in strict accordance with the Guidelines for the Care and Use of Laboratory Animals in Wuhan, Hubei, China. In addition, the investigations involving clinical cases were carried out with the informed consent of the owners of the three participating farms.

Following the death or presentation of severe clinical signs, such as emaciation, depression, and neurological deficits (including ataxia, paresis, and recumbency), in four index animals (designated Cases 1–4), postmortem examinations were performed. Gross pathological lesions suggestive of EBL, such as enlarged lymph nodes and tumor masses in the lungs and intestines, were observed. The detailed information of four cases was shown in Table S1.

During necropsy, tissue samples (lymph nodes and lung lesions) were aseptically collected from these four clinical cases. Concurrently, anticoagulated whole blood samples were collected from the general population on these farms for BLV screening, resulting in a total of 255 blood samples (Farm A: *n* = 78, Farm B: *n* = 24, Farm C: *n* = 153). The blood samples (*n* = 255) were collected using a convenience sampling method during clinical investigations to assess the viral burden within the herds following the index clinical cases, subject to farmer availability and consent.

All tissue and blood samples were transported to the Ruminant Pathogen Laboratory at Huazhong Agricultural University for analysis. Tissue samples for molecular analysis were stored at −80°C, while duplicate tissue samples were fixed in 10% neutral buffered formalin for histopathological examination.

#### 2.2.2. Pathological and Histopathological Examination

Tissue samples collected during necropsy (including lymph nodes and lung lesions) were fixed in 10% neutral buffered formalin for a minimum of 48 h to ensure adequate preservation. Following fixation, histopathological sections were prepared by a biological company (Servicebio Technology Co., Ltd., Wuhan, China).

All histopathological slides were systematically examined by an experienced veterinary pathologist who was blinded to the molecular results. The diagnosis of lymphosarcoma was confirmed on the basis of the characteristic effacement of normal tissue architecture by a monomorphic population of neoplastic lymphocytes. Cytological features such as cell size, nuclear morphology, the presence of mitotic figures, and the degree of cytoplasmic vacuolation were assessed.

### 2.3. Molecular Detection and Genotyping

#### 2.3.1. Nucleic Acid Extraction

Genomic DNA was extracted from all collected anticoagulated whole blood samples and tissue samples via the Tiangen Biotech Blood/Cell/Tissue Genomic DNA Extraction Kit (DP304, Tiangen) according to the manufacturer’s instructions. All the DNA samples were stored at −20°C until further analysis.

#### 2.3.2. Real‐Time PCR for BLV Detection

The detection of BLV proviral DNA was performed via a TaqMan‐based real‐time PCR assay targeting a conserved region of the pol gene. The reaction was carried out in a 20 μL mixture containing 10 μL of 2 × T5 Fast qPCR Mix, 0.4 μM of each primer (forward: 5^′^‐CCTCAATTCCCTTTAAACTA‐3^′^; reverse: 5^′^‐CTACCGGGAAGACTGGATTA‐3^′^), 0.2 μM of the FAM‐labeled probe (5^′^‐FAM‐CAACGCCTCCAGGCCCTTCA‐BHQ1‐3^′^), and 2 μL of template DNA. The amplification protocol comprised initial denaturation at 95°C for 5 min, followed by 50 cycles of 95°C for 20 s and combined annealing/extension at 60°C for 50 s. Each run included nuclease‐free water as a negative control and DNA from a known BLV‐positive sample as a positive control. The cycle threshold (Cq) cut‐off for a positive result was set at ≤40.95, as stipulated by the World Organization for Animal Health (WOAH) diagnostic manual for this specific assay [13]. A sample was considered positive if it yielded a characteristic amplification curve with a cycle threshold (Cq) value of ≤40.95.

#### 2.3.3. Nested PCR for Genotyping

BLV‐positive samples were further characterized by sequencing a segment of the env gene via a nested PCR protocol, as described in the WOAH diagnostic manual [13]. The first round of amplification was performed via the outer primers Outer‐F (5^′^‐CCTCCTACCAATTCTAAAGACC‐3^′^) and Outer‐R (5^′^‐CACGCAGAAGCGACAATCTC‐3^′^) under the following conditions: initial denaturation at 95°C for 3 min; 25 cycles of 95°C for 15 s, 55°C for 15 s, and 72°C for 30 s; and a final extension at 72°C for 5 min. Subsequently, 2 μL of the first‐round PCR product was used as the template for the second round of amplification with the inner primers Inner‐F (5^′^‐GGGCGGAGAAACACCYAAGG‐3^′^) and Inner‐R (5^′^‐CACTGACTATTCCACTAAGCC‐3^′^). The second‐round protocol was identical to the first, except that the annealing temperature was increased to 61°C and the number of cycles was increased to 30. The resulting amplified products were then purified and sent for Sanger sequencing to determine the BLV genotype.

#### 2.3.4. Sequencing and Phylogenetic Analysis

The amplified nested PCR products were purified and subjected to Sanger sequencing in both directions by a commercial service (Tsingke Biotechnology Co., Ltd., Beijing, China). The obtained nucleotide sequences were assembled and edited via software such as BioEdit. For genotyping, the sequences were aligned with reference BLV sequences representing known genotypes retrieved from the GenBank database via ClustalW. A phylogenetic tree was constructed via the maximum likelihood method based on the Tamura–Nei model in MEGA software (Version 11.0). The genotype of the detected BLV strains was determined by their clustering with reference sequences.

## 3. Results

### 3.1. Global BLV Prevalence and Temporal Trends

**Figure 1:**
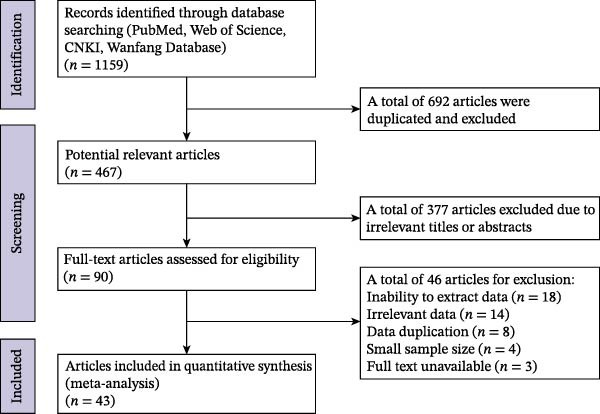
Flow chart of eligible articles for searching and selecting.

**Figure 2:**
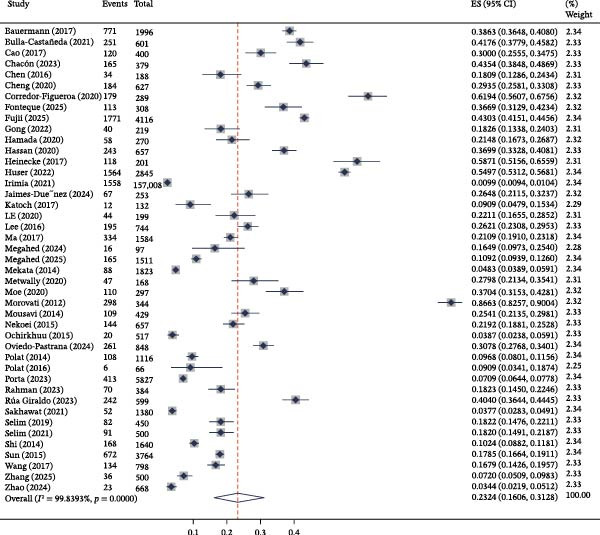
Forest plot of global pooled EBL prevalence.

**Figure 3:**
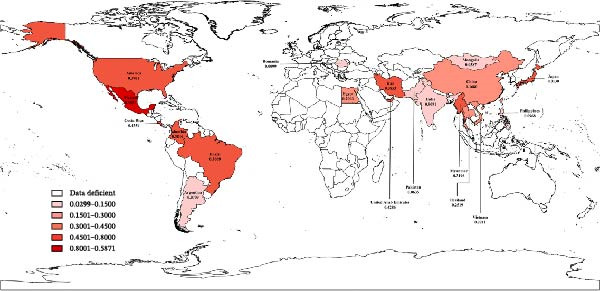
Map of global EBL prevalence.

Our systematic search and selection process ultimately identified 44 eligible studies published between 2010 and 2025 for inclusion in the meta‐analysis, providing data from 19 countries across five continents (Figure 1 and Table 1). The quality assessment indicated that 29 studies were of high quality, 11 were of moderate quality, and three were of low quality (scoring 5 points or less on the NOS scale); the inclusion of the latter two did not significantly alter the overall estimate upon sensitivity analysis (see below) (Table 1). The pooled global prevalence of BLV infection, derived from these existing reported studies, was estimated to be 23.24% (95% CI: 16.06%−31.28%), with significant heterogeneity among studies (*I* ^2^≈ 100%) (Figure 2). Geographically, the prevalence varied considerably, with the highest estimate (>60%) found in Columbia and Iran and the lowest (<10%) in countries such as Argentina, Pakistan, Mongolia, Romania, and the Philippines (Figure 3).

**Table 1:** Characteristics of studies regarding the BLV prevalence.

Author (year)	Continent	Sample type	Detection method	Positive samples	Total samples	Prevalence (%)	Quality score
Bauermann (2017)	North America	Serum	ELISA	771	1996	38.63	7
Bulla‐Castañeda (2021)	South America	Serum	ELISA	251	601	41.76	9
Cao (2017)	Asia	Serum	ELISA	120	400	30.00	5
Chacón (2023)	North America	Serum	ELISA	165	379	43.54	9
Chen (2016)	Asia	Serum	ELISA	34	188	18.09	7
Cheng (2020)	Asia	Serum	ELISA	184	627	29.35	9
Corredor‐Figueroa (2020)	South America	Whole blood	PCR	179	289	61.94	9
Fonteque (2025)	South America	Whole blood	PCR	113	308	36.68	9
Fujii (2025)	Asia	Serum	ELISA	1771	4116	43.03	5
Gong (2022)	Asia	Whole blood	PCR	40	219	18.26	7
Hamada (2020)	Africa	Whole blood	PCR	58	270	21.48	6
Hassan (2020)	Asia	Serum	ELISA	243	657	36.99	9
Heinecke (2017)	South America	Whole blood	PCR	118	201	58.71	8
Huser (2022)	North America	Serum	ELISA	1564	2845	54.97	7
Irimia (2021)	Europe	\^a^	\^a^	1558	157,008	0.99	4
Jaimes‐Due˜nez (2024)	South America	Whole blood	PCR	67	253	26.48	9
Katoch (2017)	Asia	Serum	ELISA	12	132	9.09	8
LE (2020)	Asia	Serum	ELISA	44	199	22.11	9
Lee (2016)	Asia	Whole blood	PCR	195	744	26.21	7
Ma (2017)	Asia	Serum	ELISA	334	1584	21.09	10
Megahed (2024)	North America	Serum	ELISA	16	97	16.49	7
Megahed (2025)	North America	Serum	ELISA	165	1511	10.92	7
Mekata (2014)	Asia	Serum	ELISA	88	1823	4.83	8
Metwally (2020)	Africa	Whole blood	PCR	47	168	27.98	8
Moe (2020)	Asia	Whole blood	PCR	110	297	37.04	8
Morovati (2012)	Asia	Serum	ELISA	298	344	86.63	9
Mousavi (2014)	Asia	Serum	ELISA	109	429	25.41	8
Nekoei (2015)	Asia	Whole blood	PCR	144	657	21.92	7
Ochirkhuu (2015)	Asia	Whole blood	PCR	20	517	3.87	7
Oviedo‐Pastrana (2024)	South America	Serum	ELISA	261	848	30.78	8
Polat (2014)	Asia	Whole blood	PCR	108	1116	9.68	9
Polat (2016)	Asia	Whole blood	PCR	6	66	9.09	8
Porta (2023)	South America	Serum	ELISA	413	5827	7.09	7
Rahman (2023)	Asia	Serum	ELISA	70	384	18.23	8
Rúa Giraldo (2023)	South America	Serum	ELISA	242	599	41.13	8
Sakhawat (2021)	Asia	Serum	ELISA	52	1380	3.77	9
Selim (2019)	Africa	Serum	ELISA	82	450	18.22	9
Selim (2021)	Africa	Serum	ELISA	91	500	18.20	9
Shi (2014)	Asia	Serum	ELISA	168	1640	10.24	8
Sun (2015)	Asia	Serum	ELISA	672	3764	12.41	9
Wang (2017)	Asia	Whole blood	PCR	134	798	16.79	8
Zhang (2025)	Asia	Serum	ELISA	36	500	7.20	9
Zhao (2024)	Asia	Whole blood	PCR	23	668	3.44	9

^a^\ indicates that the original article did not clearly specify the sample type and detection method.

A critical observation was a marked temporal shift in reported prevalence. The pooled prevalence from studies published before 2018 was 20.31%, which dropped sharply to 4.35% in studies published after 2018 (Table 2). However, meta‐regression analysis indicated that this downward trend was not influenced by temporal effects (*p* = 0.350), and the sustained decline was concentrated within pooled global data rather than occurring in any specific region (*p* = 0.156). Sensitivity analysis, performed via the leave‐one‐out method, confirmed that the overall pooled estimate was robust and not disproportionately influenced by any single study (Figure S1).

**Table 2:** Global pooled prevalence of EBL according to different related factors.

Subgroup and item		Events	Total	Positive rate (95% CI)	*Q*	*P*	*I* ^2^ (%)	*p*‐Value^a^
Regions	Asia	5015	23,249	0.2141 (0.2088–0.2194)	3492.22	<0.0001	99.31	0.19
	South America	1644	8926	0.1842 (0.1762–0.1924)	1007.24	<0.0001	99.60	
	North America	2681	6828	0.3926 (0.3810–0.4043)	1506.01	<0.0001	99.54	
	Africa	278	1388	0.2003 (0.1795–0.2223)	8.34	<0.0001	64.02	
Publication year	Before 2018	3331	16,399	0.2031 (0.1970–0.2094)	2074.56	<0.0001	99.23	0.79
	After 2018	7845	181,000	0.0433 (0.0424–0.0443)	18782.55	<0.0001	99.87	
Sample type	Serum	8256	33,820	0.2441 (0.2395–0.2487)	6180.36	<0.0001	99.58	~0.001
	Whole blood	1362	6571	0.2073 (0.1975–0.2173)	873.88	<0.0001	99.43	
Detection method	ELISA	8256	33,820	0.2441 (0.2395–0.2487)	6180.36	<0.0001	99.58	~0.001
	PCR^b^	1362	6571	0.2073 (0.1975–0.2173)	873.88	<0.0001	99.43	
Breed	Dairy cow	3426	11,499	0.3014 (0.2930–0.3099)	3291.21	<0.0001	99.51	0.52
	Beef	2773	10,811	0.2565 (0.2483–0.2648)	1561.33	<0.0001	99.42	
Gender	Female	644	1727	0.3729 (0.3500–0.3962)	545.35	<0.0001	99.08	0.19
	Male	767	4180	0.1835 (0.1719–0.1956)	203.72	<0.0001	96.56	
Age	>12 months	2887	9425	0.3063 (0.2970–0.3157)	1734.40	<0.0001	99.48	0.02
	≤12 months	283	1895	0.1493 (0.1336–0.1662)	32.13	<0.0001	84.44	

^a^The *p*‐value represents the statistical difference value between variables within each subgroup analysis, and when the *p*‐value less than 0.05 indicates that comparisons between variables in that subgroup are statistically significant.

^b^PCR method, including PCR, real‐time PCR, and nested PCR.

### 3.2. Key Epidemiological Factors Influencing BLV Prevalence

Subgroup analyses revealed several factors significantly associated with the prevalence of BLV infection. Sample type and detection method significantly influenced detection rates. ELISA testing of serum samples yielded a seroprevalence (24.41%) that showed a statistically significant difference compared to PCR performed on whole blood (20.65%) (*p* < 0.01). Another pronounced difference was observed by age, where cattle more than 12 months presented a much higher prevalence (30.63%) than calves less than 12 months did (14.93%) (*p* < 0.05). The production type was also a major factor. Specifically, to address the epidemiological differences between production systems, we analyzed dairy and beef cattle separately. The results showed that dairy herds had a higher infection rate (30.14%) than beef herds did (25.65%), highlighting the necessity of considering these two groups as distinct populations. Furthermore, female cattle had a much higher prevalence (37.29%) than males did (18.35%). A summary of the subgroup analyses is provided in Table 2.

Subsequently, a multiple meta‐regression model was constructed to further evaluate the potential sources of heterogeneity, and the age group and the detection methods (sample type) groups that showed significant differences in the subgroup analysis were included as covariates. The results indicated that the detection method was a significant predictor of the reported prevalence. Specifically, studies using the ELISA method reported a statistically difference in prevalence (coefficient = −3.245, *p* = 0.003), which was not observed in studies using PCR method (coefficient = −0.665, *p* = 0.849). In contrast, age grouping was not identified as a significant source of heterogeneity in the multifactor model. After adjusting for the influence of the detection method, neither the subgroup with cattle more than 12 months (coefficient = 0.612, *p* = 0.140) nor the subgroup with calves less than 12 months (coefficient = −0.127, *p* = 0.809) showed a statistically significant association with prevalence.

### 3.3. The Chinese Context: National Estimate Masks a Severe Localized Resurgence in Hubei

The pooled BLV prevalence for China, calculated from the meta‐analysis, was 16.80% (Figure 3), fluctuating over the observation period from 2010 to 2025. Although these data originated from Northeast China, Northwest China, Henan Province, Shanghai City, Guiyang City, and Sanming City, they did not cover the entire country; thus, this average concealed regional heterogeneity within the country, with reported rates ranging from low endemicity to hyperendemic foci [9].

Despite this moderate national figure and the apparent global post‐2018 decline, our targeted investigation in Hubei Province revealed an alarming situation. Real‐time PCR screening of 255 cattle across three farms demonstrated an overall BLV positivity rate of 49.41% (126/255). This high burden was driven predominantly by two farms: Farm B presented a positivity rate of 62.5% (15/24), and Farm C reached 66.7% (102/153), indicating intense within‐herd transmission.

### 3.4. Clinical and Pathological Confirmation of EBL in Hubei Patients

**Figure 7:**
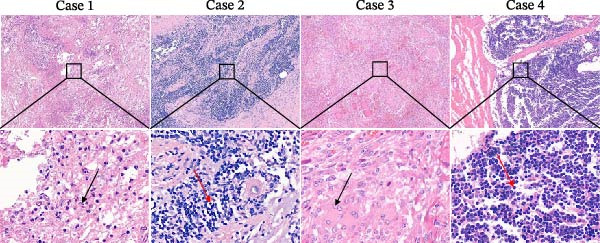
Histopathological images of Cases 1–4 by H&E. Cases 1, 2 and 4 are lymph node tissue samples, and Case 3 is a lung lesion mass. The scale sizes are 100 μm (top) and 20 μm (bottom), respectively. The figure below is an enlargement of part of the area of the upper figure. The black arrow indicates the mitotic figures, while the red arrow points to the bare nuclei.

The high infection rate in Hubei Province was directly linked to clinical disease, as confirmed by the investigation of four fatal cases (Cases 1–4) from Farms A and B. Postmortem examinations revealed gross lesions pathognomonic for EBL, including severe enlargement of lymph nodes (mediastinal, pulmonary, and mesenteric) and multiple tumor masses in the lungs and intestines (Figures 4–6). Histopathological analysis confirmed the diagnosis of lymphosarcoma, demonstrating effacement of normal tissue architecture by densely packed, pleomorphic neoplastic lymphocytes with scant cytoplasm and frequent mitotic figures (Figure 7).

Figure 4Pathological examination in Cases 1 and 2. (A) Pulmonary lymph node enlargement in Case 1, (B) tumor masses located in the intestinal region in Case 2, (C) the cross‐section of the diseased mesenteric lymph nodes in Case 2. The arrow indicates the tumor mass, and the circle marks the presence of pus.

**Figure:**
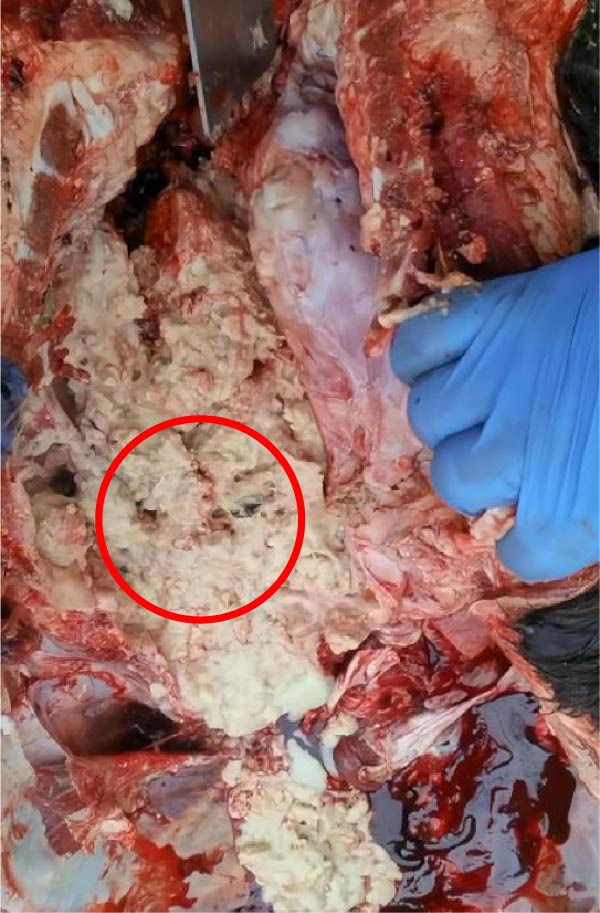
(A)

**Figure:**
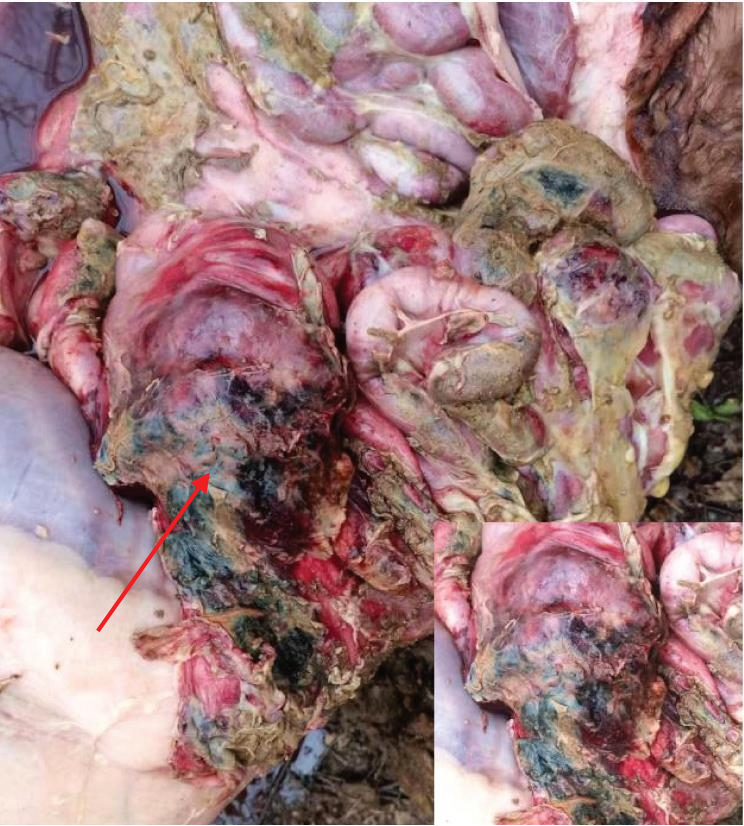
(B)

**Figure:**
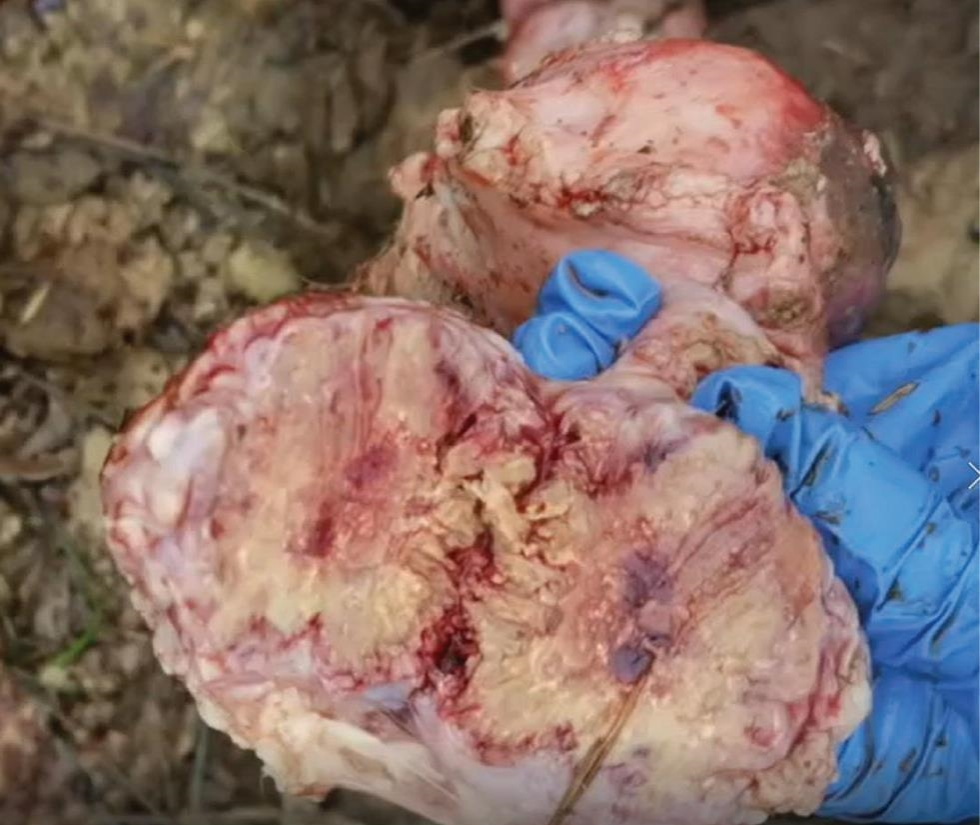
(C)

Figure 5Pathological examination in Case 3. (A) Gross lesions in the lungs, (B) adhesion between the lung and pleura, (C) large tumor masses aggregated on the lung surface, and (D) dispersed tumor masses on the lung surface. The arrow points to the tumor mass, while the circle indicates the site of adhesion between the lung and the pleural cavity.

**Figure:**
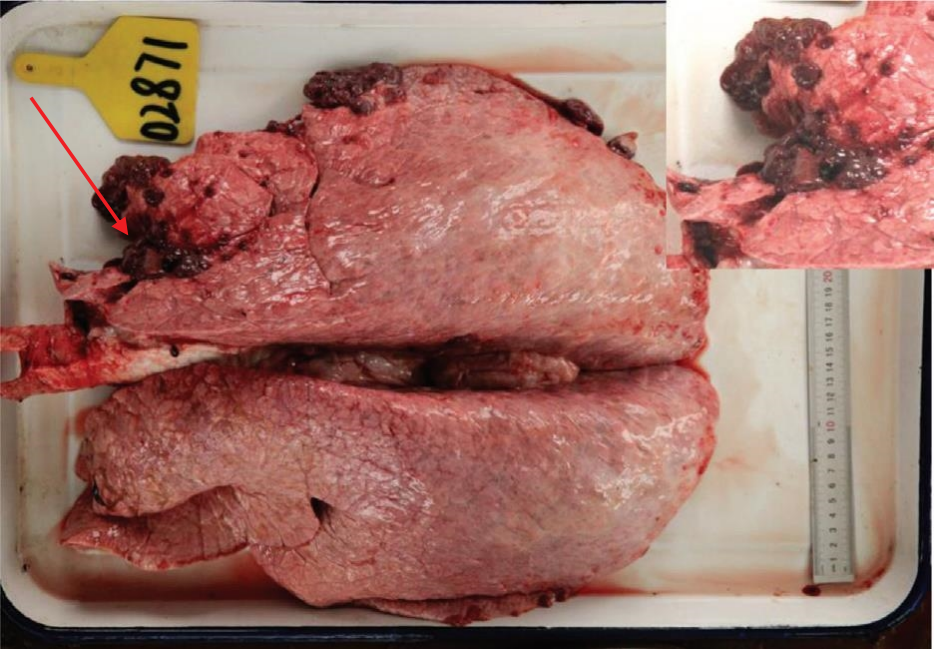
(A)

**Figure:**
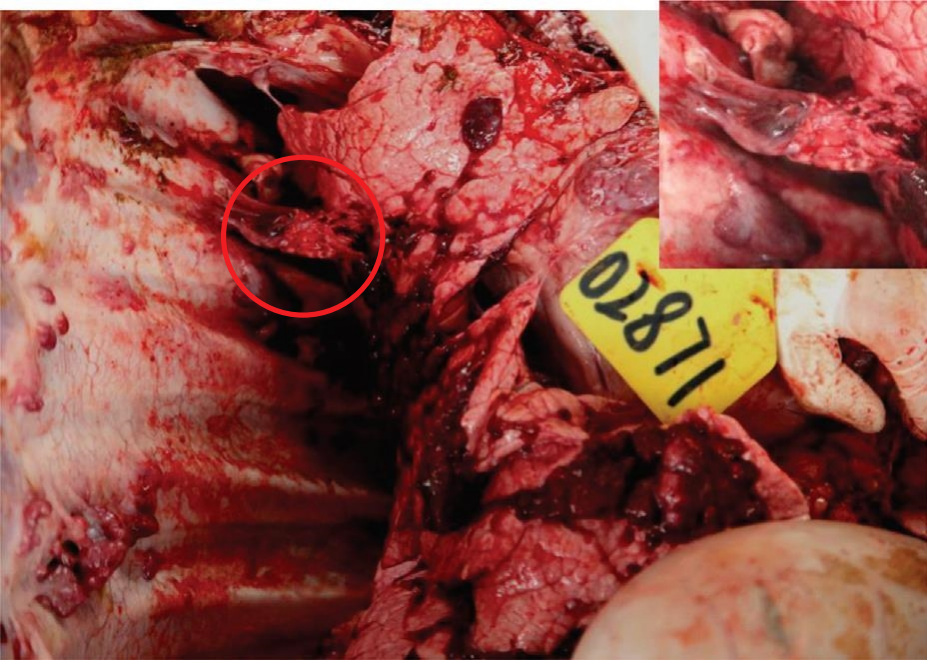
(B)

**Figure:**
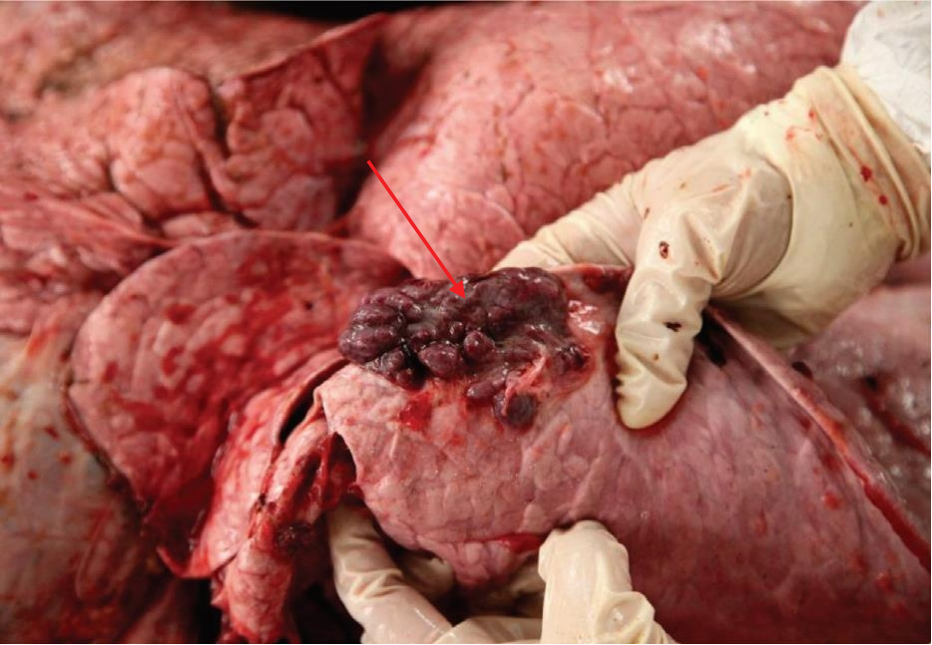
(C)

**Figure:**
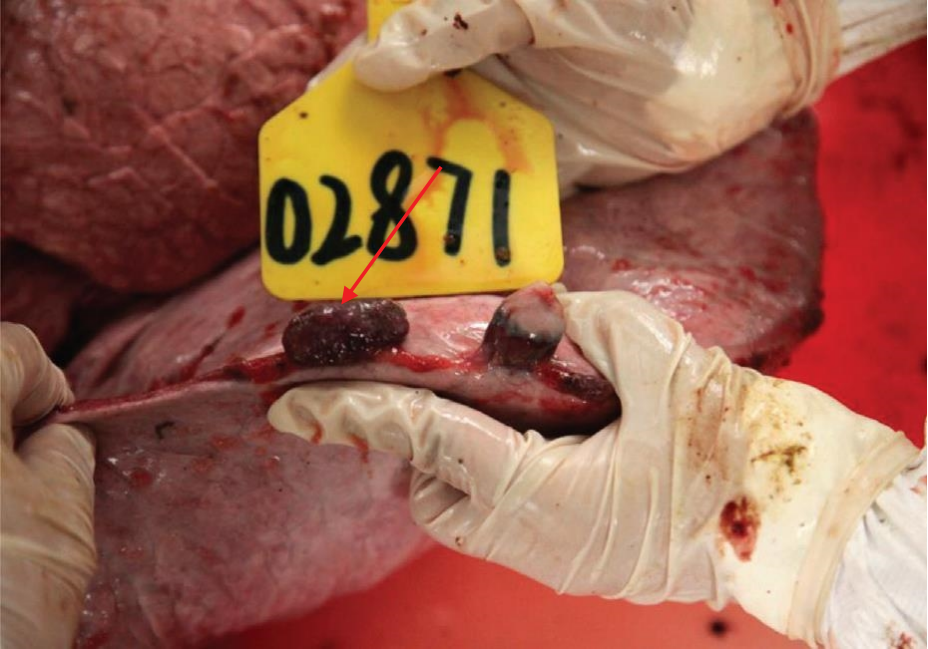
(D)

Figure 6Pathological examination in Case 4. (A) Tumor masses within the lung, (B) tumor masses on the lung surface, (C) thoracic tumor mass, and (D) enlargement of the hilar lymph node, resembling the size of an egg. The arrow indicates the tumor mass, and the circle marks the enlarged hilar lymph nodes.

**Figure:**
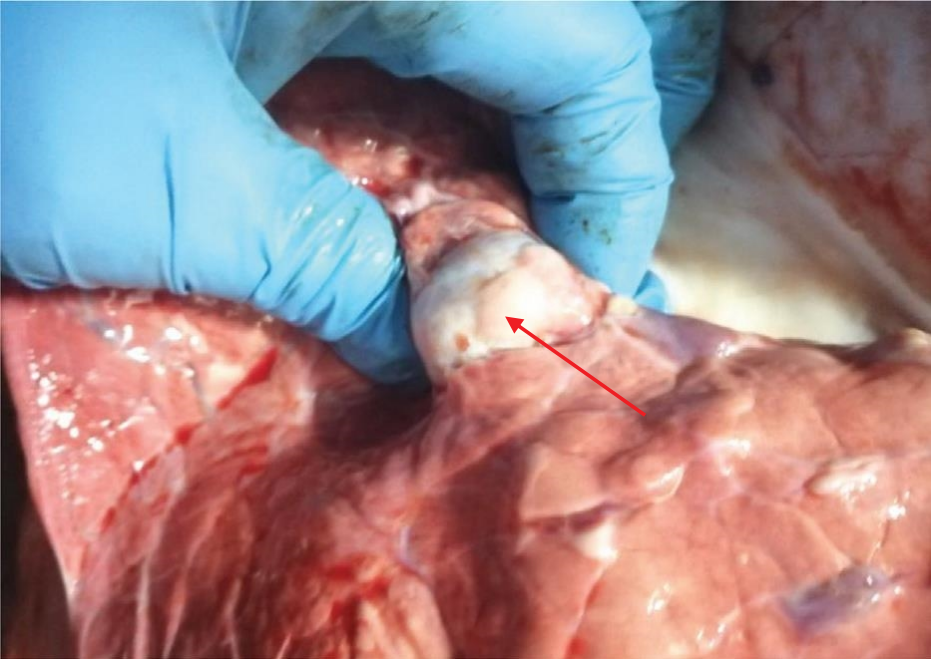
(A)

**Figure:**
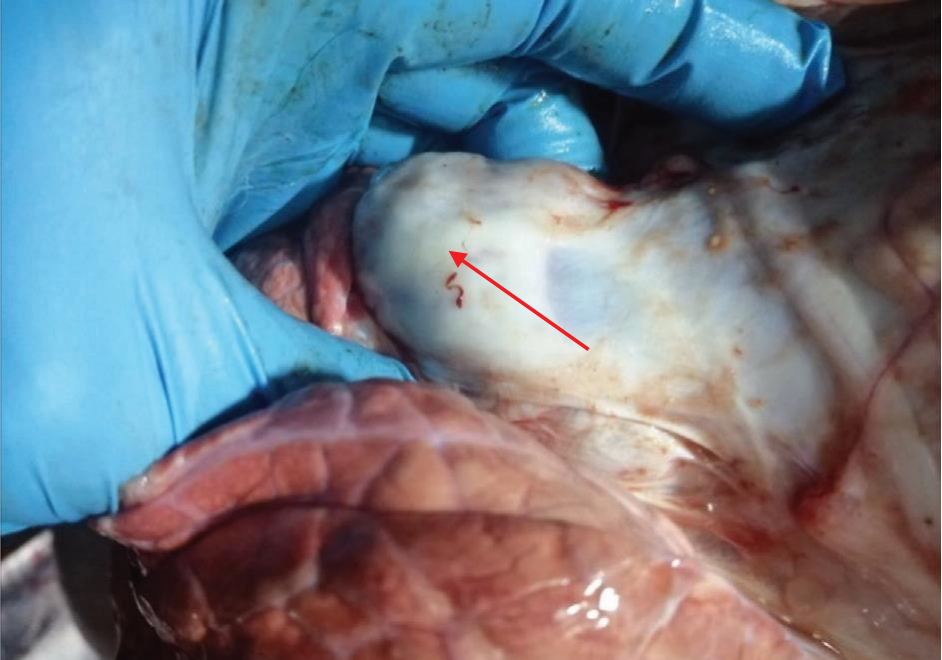
(B)

**Figure:**
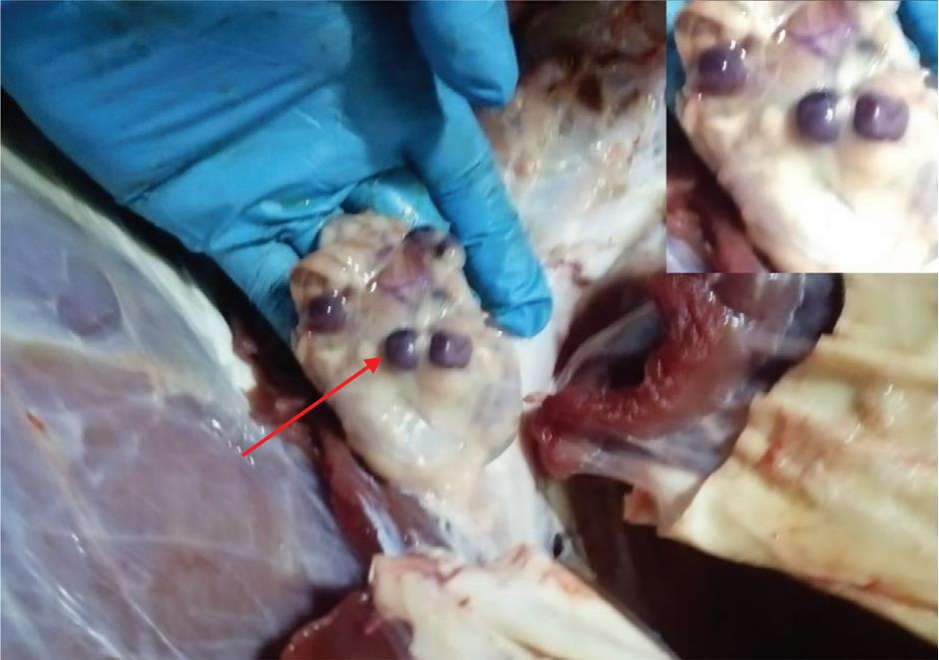
(C)

**Figure:**
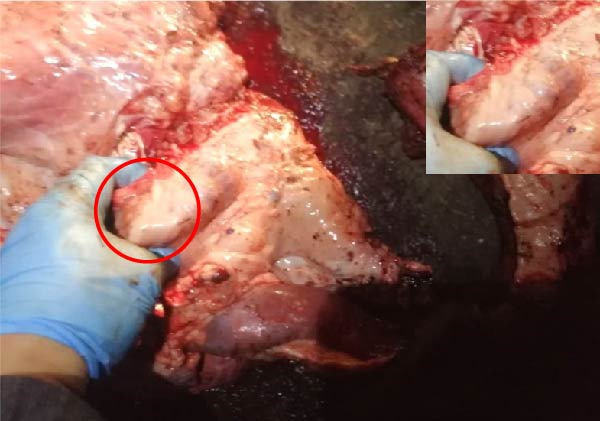
(D)

**Figure 8:**
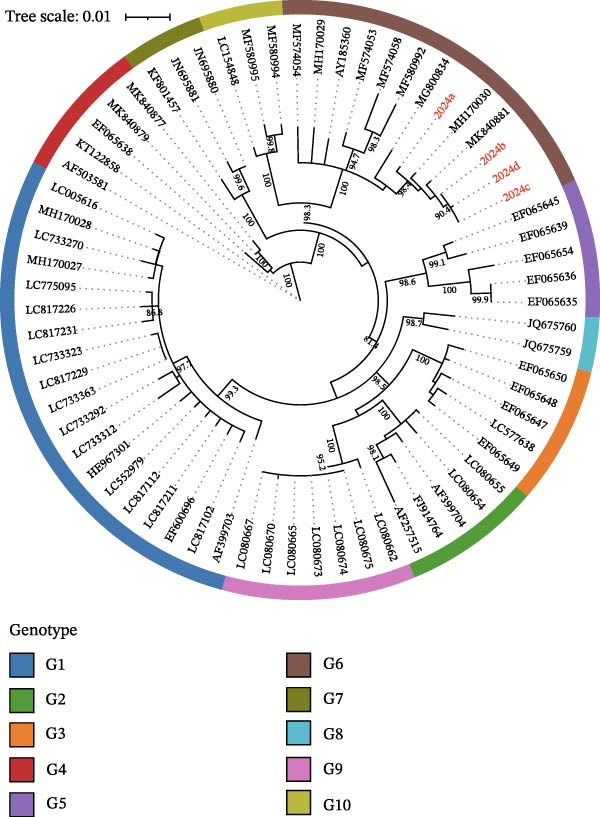
Phylogenetic analysis based on full‐length env gene sequences. BLV isolates from this study are indicated in red. 2024a, 2024 b, 2024c, and 2024d corresponded Case 1, Case 2, Case 3, and Case 4, respectively.

Molecular testing confirmed the presence of BLV proviral DNA in all tissue samples from these clinical cases via real‐time PCR. Subsequent genotyping by nested PCR and sequencing of the env gene identified the infecting virus in all four cases as belonging to the G6 genotype (Figure 8).

## 4. Discussion

This integrated study, which combines a global meta‐analysis with field‐based case investigations, offers a comprehensive overview of the current BLV landscape. Our findings reveal two contrasting narratives: a potential decline in pooled BLV prevalence based on accessibly updated data in recent years and a severe and ongoing resurgence in a major Chinese cattle‐farming region.

The pooled global BLV prevalence based on updated known data of 23.24%, confirming that the virus remains a pervasive significant pathogen worldwide. The substantial heterogeneity observed is expected, reflecting differences in control policies, farming practices, and surveillance efforts across countries. While extremely high heterogeneity was predictable, “pooled global BLV prevalence based on the systematic analysis of existing reported studies” seems to be a more appropriate and cautious description of this result rather than “pooled global prevalence” under such extreme conditions.

Methodological quality represents a potential source of bias in meta‐analyses. The majority of studies included in this research were of high quality, and sensitivity analyses further confirmed that the inclusion of a small number of low‐quality studies (*n* = 3) did not substantially alter the pooled prevalence estimates. This stability indicates that the observed heterogeneity is more likely attributable to ecological and biological factors rather than methodological shortcomings in the original sources. Subgroup analysis and multiple regression analysis identified that sample type and detection method were the methodological factors associated with significant heterogeneity in the reported prevalence. Serological testing reflected the immune response to BLV. Once established, antibodies persisted long‐term in the host even when viral load was extremely low or undetectable by nucleic acid assays during some phases, which indicated that serological testing better reflects the cumulative degree of past infection [14]. This phenomenon of serological positivity with negative PCR results was not uncommon: A herd study demonstrated that although the ELISA antibody positivity rate reached 55% (1564/2845), only 53.6% (838/1564) of these positive individuals tested positive by qPCR [15]. Therefore, considering the cost‐effectiveness and operational convenience of large‐scale monitoring, it is recommended to use serological testing for initial screening to determine the cumulative number of exposed animals. Subsequently, nucleic acid assays can be retested to assist in epidemiological analysis and prevention strategies [16]. Another pivotal factor influencing the pooled prevalence is the production type. Subgroup analyses clearly differentiated these populations to address potential representativeness biases. Results showed a significantly higher prevalence rate in dairy herds (30.14%) compared to beef cattle herds (25.65%), aligning with the well‐established epidemiology of BLV [3, 17]. The relatively longer productive lifespan of dairy cows compared to beef cattle (which are often slaughtered at a younger age), and management practices that facilitate iatrogenic transmission create an ideal environment for sustained viral spread within herds [18]. Although dairy cows and beef cattle belong to different epidemiological populations, combining their data for analysis can provide a comprehensive estimate of the overall burden of the species. Excluding either population introduces selection bias, thereby compromising the validity of pooled prevalence estimates. We also acknowledge that stratified analysis is crucial for accurately interpreting the global situation. Reliance solely on pooled estimates may obscure these differences, so a comprehensive and integrated understanding of the global BLV epidemiology is necessary.

Another finding is the apparent decline in reported prevalence based on the available data in BLV prevalence in studies published after 2018 (4.35%) compared with those published before 2018 (20.31%). This phenomenon might stem from updated data from countries with low prevalence, all of which are derived from large‐sample datasets [19–21], it was possible that the pooled BLV prevalence did not truly represent a global improvement, so this result should be interpreted with caution. Although the large scale of these studies might bias the pooled estimate downward, incorporating these data is crucial for avoiding selection bias and accurately reflecting the success of eradication in certain regions. Moreover, despite geographical distribution imbalances, this trend indeed reflected some genuine progress, attributable to successful eradication programs in several developed countries, increased farmer awareness, and improved biosecurity measures [22, 23]. However, this global picture is contradicted by the situation uncovered in Hubei Province, China.

In China, the pooled prevalence of BLV was found to be 16.80%. However, these samples did not cover the entire country, thus, this figure still masks regional disparities. Our surveillance data indicated a high positive rate of 49.41% among cattle herds from three farms in Hubei Province, exceeding the average aggregated estimate of China based on the reported data, which further suggested potential geographic imbalance in the pooled prevalence. This high positive rate also coincided with the historical data, reporting a prevalence of 49.11% in high‐burden regions [9]. Although the reported prevalence of 49.41% in this study might be more applicable to high‐risk herds or regions, this finding still indicated a resurgence of bovine leukemia in Hubei Province. Future studies should incorporate herd‐based random sampling strategies to obtain more accurate and representative estimates of the overall prevalence.

This high positivity rate sharply raises concerns about the localized resurgence of BLV in China, which may be attributed to a “control‐effort gap.” While many developed countries have implemented successful, systematic eradication programs underpinned by stringent policies and financial compensation [3, 24], control strategies in high‐prevalence regions of China might still rely heavily on fragmented, voluntary measures [8]. This gap could be exacerbated by factors such as high animal density, long‐distance cattle movement without rigorous testing, and insufficient on‐farm biosecurity protocols, creating a perfect storm for BLV persistence and spread [18, 23, 25]. Moreover, according to the epidemiological investigation results in Hubei Province, the identification of the G6 genotype is consistent with previous reports from China, suggesting that this strain continues to persist as high‐burden endemicity hotspots [23]. The above findings demonstrate that certain important livestock production areas in Hubei Province and all high‐prevalence countries/regions may be experiencing a worsening situation due to gaps in control efforts, which underscores the necessity of complementing broad epidemiological surveillance with targeted, ground‐level monitoring to identify emerging hotspots.

Therefore, our study leads to two critical conclusions. For the global community, the “declining trend” based on the availably updated data should not lead to complacency, continued vigilance is essential. For countries/regions with high prevalence, our findings sound an urgent alarm. Immediate, targeted actions are needed, including (1) implementing enhanced and routine BLV surveillance programs, (2) enforcing strict biosecurity measures to prevent iatrogenic transmission, (3) considering test‐and‐segregation or culling strategies for infected animals in valuable dairy herds, and (4) launching educational initiatives for farmers and veterinarians about the economic impact and transmission routes of BLV [3, 24]. In the evolving global BLV landscape, control efforts should be sustained in regions showing progress and launching decisively, while targeted interventions should be implemented in all high‐risk countries/regions to curb active localized resurgence.

This study has several limitations that should be considered when interpreting the results. Although meta‐analyses of cross‐sectional studies/continuous variables typically exhibit high heterogeneity, the substantial heterogeneity observed during the study process still imposes certain restrictions on the accuracy of the overall estimations. Moreover, differences in study design, sampling strategies, diagnostic methods, and geographic imbalances among included studies introduced potential biases, which also constrained the ability to analyze important confounding factors. It should be noted that these results represent a synthesized analysis based on reported data and do not constitute a definitive global epidemiological indicator. Third, the sample sizes included in the study varied considerably, ranging from investigations of small populations to large‐scale surveillance programs. Although a random‐effects model was employed to limit the statistical weighting of larger studies and sensitivity analysis was conducted to ensure robust results, the global prevalence estimate should be interpreted with caution. This data integrates complex regional realities, encompassing both eradicated areas and high‐prevalence hotspots, rather than reflecting a uniform global baseline. Finally, the multivariate meta‐regression analysis indicates that detection methods significantly influence prevalence estimates, with the ELISA method yielding higher results than the PCR method. Given that most samples included in this meta‐analysis employed ELISA methods, this imbalance may lead to an upward bias in the pooled global prevalence estimate. Consequently, the reported data should be understood that it may primarily reflects serological exposure history.

## 5. Conclusion

In conclusion, this study delineates two counter‐trending phenomena for BLV: an apparent decline in reported prevalence based on the available data post‐2018 stands in sharp contrast to a severe resurgence in part of the Hubei Province, China, where an alarming 49.41% infection rate and confirmed clinical cases were documented. This stark disparity serves as a crucial warning that national and global averages can mask dangerous local epidemics, demanding immediate, targeted interventions in high‐risk regions to mitigate the ongoing threat.

## Author Contributions

**Ruicheng Yu and Sen Zhang**: conceptualization, formal analysis, investigation, methodology, resources, software, visualization, writing – original draft. **Zhijie Xiang and Liyun Wan**: formal analysis, investigation, methodology, resources. **Marawan A. Marawan and Jianguo Chen**: conceptualization, project administration, supervision. **Aizhen Guo and Yingyu Chen**: conceptualization, funding acquisition, project administration, supervision, writing – review and editing.

## Funding

This work was supported by the National Key Research and Development Program of China (Grant 2022YFD1800702) and the China Agriculture Research System of MOF and MARA (Grant CARS‐37).

## Ethics Statement

This animal experiment was approved by the Animal Experiment Ethics Committee of Huazhong Agricultural University (Approval Number: HZAUCA‐2024‐0036). All procedures were conducted in strict accordance with the Guidelines for the Care and Use of Laboratory Animals in Wuhan, Hubei, China. In addition, the investigations involving clinical cases were carried out with the informed consent of the owners of the three participating farms.

## Conflicts of Interest

The authors declare no conflicts of interest.

## Supporting Information

Additional supporting information can be found online in the Supporting Information section.

## Supporting information


**Supporting Information** The detailed information of four cases was shown in Table S1. The results of sensitivity analysis was in Figure S1.

## Data Availability

The data supporting the findings of this study are available within the article. Further inquiries can be directed to the corresponding authors.
