# Supplementary material for: Bovine Leukemia Virus: Global Prevalence and Localized Resurgence in China Revealed by an Integrated Meta‐Analysis and Case Series
Source: Transbound Emerg Dis. 2026 Apr 10;2026:2598815. doi: 10.1155/tbed/2598815 (PMC13066710; doi:10.1155/tbed/2598815)
Supplement: Supplementary file 1 — Supporting Information The detailed information of four cases was shown in Table S1. The results of sensitivity analysis was in Figure S1. [file TBED-2026-2598815-s001.docx]

**Table S1.** Key information for the four clinical cases.

| Case No. | Breed | Age Category | Sex | Production Status | Main Clinical Signs |
| --- | --- | --- | --- | --- | --- |
| Case1 | Simmental cattle | Adult | Male | Beef cattle  (fattening stage) | Weight loss, emaciation, hindlimb lameness, dyspnea |
| Case2 | Simmental cattle | Adult | Male | Beef cattle  (fattening stage) | Weight loss, emaciation, hindlimb lameness, diarrhea, ataxia |
| Case3 | Holstein cattle | Calf | Female | Growing stage | Tachypnea, emaciation, lethargy, reduced appetite |
| Case4 | Holstein cattle | Heifer | Female | Replacement heifer  (not yet bred or calved) | Corneal opacity, emaciation, circling, hindlimb lameness, rough hair coat, recumbency |





**Figure S1.** Results of sensitivity analysis using the leave-one-out cross-validation method.
